# Arg‑C Ultra Simplifies Histone Preparation for LC-MS/MS

**DOI:** 10.1021/acs.analchem.5c02238

**Published:** 2025-06-12

**Authors:** Palina Ryzhaya, Pavlína Pírek, Zbyněk Zdráhal, Gabriela Lochmanová

**Affiliations:** † Mendel Center for Plant Genomics and Proteomics, Central European Institute of Technology, 37748 Masaryk University, 625 00 Brno, Czech Republic; ‡ National Centre for Biomolecular Research, Faculty of Science, Masaryk University, 625 00 Brno, Czech Republic

## Abstract

Arginine-specific cleavage is the primary method used to prepare lysine-rich histone proteins in bottom-up proteomics. As the Arg-C enzyme has demonstrated suboptimal specificity, cleavage at the carboxyl side of arginine residues is typically achieved through the chemical derivatization of lysines followed by trypsin digestion. Recent improvements in proteolytic enzymes are reflected in the introduction of Arg-C Ultra, a recombinant proteinase with a substantially improved digestion specificity. Here, using mammalian histone extract, we demonstrate that Arg-C Ultra facilitates histone preparation for LC-MS/MS. We show the performance of Arg-C Ultra in terms of digestion specificity, number of modified forms identified, and yield of quantitative information compared with Arg-C and trypsin digestion combined with chemical derivatization with trimethylacetic anhydride. Importantly, we show that chemical derivatization at the peptide level, i.e., after Arg-C Ultra digestion, is still necessary to improve the quantification of short histone peptidoforms as well as positional isomers.

## Graphical abstract

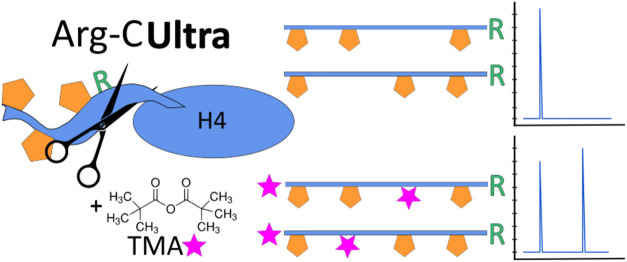

## Introduction

The diversity conferred by the chemical structure of histones is a hallmark of eukaryotic chromatin, allowing the dynamic regulation of DNA replication, transcription, and repair. The chromatin of eukaryotic organisms exhibits a range of histone variants with distinct amino acid sequences.^1^ Together with frequently occurring post-translational modifications (PTMs) at different amino acid residues, they define distinct chromatin states determining specific functions.^2^ PTMs are not limited to acetylation, methylation, or phosphorylation, but many other rare PTMs have been identified in histones in specific tissues, at different developmental stages, or in association with diseases.^3–6^

The enormous complexity of histone proteins makes their study difficult and predetermines the limitations of their analysis. In particular, bottom-up proteomics addresses the following quantification issues: (i) high amino acid sequence similarity between certain histone variants (e.g., 96% identity between human H3.1 and H3.3) prevents assignment of the majority of identified peptides to the original variant and (ii) lysine- and arginine-rich histones with diverse modification patterns result in the formation of short hydrophilic peptidoforms of different lengths after trypsin digestion. To partially address the former, the ratio between histone sequence variants can be determined by quantifying unique peptides.^7–9^ For the latter, chemical derivatization of amine groups has been introduced prior to trypsin digestion to prevent cleavage at the unmodified lysine residues, resulting in Arg-C-like peptides.^10^ Mimicking Arg-C digestion outweighed the simple usage of Arg-C regarding digestion site specificity and reproducibility.^11^ The second derivatization step is carried out after trypsin digestion to label the released amine groups at the N-termini of the peptides. Depending on the chemical properties of the derivatization reagent used, the increased hydrophobicity of peptides improves chromatographic retention and separation, leading to a higher number of identified histone forms. For instance, for the detection of certain hydrophilic peptides, such as the peptide containing PTMs at H3K4, a hybrid labeling procedure involving a combination of propionylation at the protein level and phenyl isocyanate labeling at the peptide level (Prop-PIC) is used instead of the conventional propionylation procedure.^10,12^ Recently introduced microwave derivatization using trimethylacetic anhydride (TMA) improves the separation of isobaric peptide forms, thereby addressing the quantification of frequently occurring histone positional isomers.^13^ The choice of reagents is limited because different chemical compounds have a major impact on the ionization efficiency of peptides; thus, they perturb, to a greater or lesser extent, the estimation of abundances of variably modified forms of the same peptide sequence.^14^ Another limitation is the size of the chemical compound as labeling with larger molecules, especially molecules with aromatic rings (i.e., PIC), is inefficient for the derivatization of lysine residues at the protein level.^12,14^ Ongoing research into the effectiveness of derivatization reagents,^10,12–14^ proteinases,^11,15^ LC-MS/MS methods,^16,17^ and software tools^15,18^ highlights the need to further improve the methodology for comprehensive histone analysis.

In the present study, we demonstrate the performance of a novel proteinase Arg-C Ultra by comparing it with Arg-C and trypsin digestion in combination with TMA derivatization. Arg-C Ultra’s enhanced digestion specificity compared to Arg-C allows its use as a time-saving alternative to histone derivatization with TMA followed by trypsin digestion. However, we show that peptide-level derivatization is required for most histone peptides after cleavage with Arg-C Ultra to achieve results comparable to the trypsin-based approach.

## Experimental Section

### Materials and Reagents

Details of materials and reagents used in this work are described in the Supporting Information.

### Preparation of Histone Extracts from Human Cell Culture

MEC-1 chronic lymphocytic leukemia cell line was used for histone preparation. Histones were extracted following the established protocol;^19^ see the Supporting Information for details.

### Chemical Derivatization of Histone Proteins before Digestion

Before trypsin digestion, 5 μg of histone extract was chemically derivatized with TMA according to a previously published procedure.^13^ Briefly, histone extract was diluted to a final concentration of 1 μg.μL^– 1^ with 50% (v/v) ACN. The pH was adjusted to 8 with NH_4_OH, and 2.25 μL of derivatization reagent consisting of TMA and ACN in a 1:3 (v/v) ratio was added to each sample. The samples were incubated for 5 h at room temperature with shaking, followed by repeated derivatization step including 16 h incubation, and then subjected to two rounds of microwave-assisted derivatization (adjustment of the sample’s pH to 8 with NH_4_OH, addition of 2.25 μL of derivatization reagent, and two 1 min incubations in the microwave oven at 350 W with a short centrifugation between them). The samples were concentrated in a Savant SPD121P concentrator (Thermo Fisher Scientific) and diluted with 50% (v/v) ACN to a final volume of 9 μL, and the second round of microwave-assisted derivatization was carried out with the same protocol.

### Digestion of Histone Extracts

For histone digestion, Arg-C (V188A; Promega, WI), Arg-C Ultra (VA1831; Promega), or SOLu-Trypsin Dimethylated (EMS0005; Merck) proteases were used.

#### Arg-C

5 μg of histone extract was dissolved in incubation buffer (50 mM Tris-HCl (pH 7.6–7.9), 5 mM CaCl_2_, and 2 mM ethylenediaminetetraacetic acid (EDTA)) to a final protein concentration of 1 μg.μL^– 1^. Arg-C protease was added at a 1:100 (w/w) enzyme-to-substrate ratio. An activation buffer (5 mM Tris-HCl (pH 7.6–7.9), 5 mM dithiothreitol (DTT), and 0.2 mM EDTA) was added. The samples were incubated at 37 °C for 2 h, and then the digestion was stopped by adding formic acid (FA) to a final concentration of 1%. The samples were dried in a vacuum concentrator and desalted.

#### Arg-C Ultra

5 μg of histone extract was combined with ammonium bicarbonate buffer (AB; pH 8.0) and DTT to final concentrations of 50 mM and 10 mM, respectively, with the protein concentration adjusted to 1 μg.μL^– 1^. Arg-C Ultra was added at a 1:100 (w/w) enzyme-to-substrate ratio. The mixture was incubated at 37 °C for 2 h. The digestion was either stopped by adding FA to a final concentration of 1%, dried in a vacuum concentrator, and desalted, or subjected to subsequent derivatization steps.

#### Trypsin

SOLu-Trypsin Dimethylated diluted in 100 mM AB (pH 8.0) was added to the TMA-labeled protein samples at a 1:40 (w/w) enzyme-to-substrate ratio, and the mixture was incubated at 37 °C for 4 h. Following the first incubation, an additional trypsin aliquot was added at the same ratio, and the samples were incubated for an additional 12 h at 37 °C. The samples were subjected to subsequent derivatization steps.

### Chemical Derivatization of Histone Peptides

A microwave-assisted derivatization with TMA, following the protocol outlined previously,^13^ was used to increase peptide hydrophobicity. The pH of each sample was adjusted to 8 using NH_4_OH. The samples were subjected to two rounds of microwave-assisted derivatization (adjustment of the sample’s pH to 8 with NH_4_OH, addition of 2.25 μL of derivatization reagent, and two 1 min incubations in the microwave oven at 350 W with short centrifugation between them). The labeled peptides were dried overnight in a vacuum concentrator and desalted.

### LC-MS/MS Analysis, Database Search, Data Evaluation, and Statistical Analysis

The samples were desalted using AttractSPE Tips C18 and analyzed using an Ultimate 3000 RSLCnano liquid chromatograph coupled to an Orbitrap Fusion Lumos Tribrid mass spectrometer (Thermo Fisher Scientific). Mass spectra were acquired in the data-dependent acquisition mode. Raw data were searched against a modified cRAP contamination database, an in-house histone human database, and the UniProt KB Human database using the in-house Mascot search engine (v2.6.2; Matrix Science, MA) via Proteome Discoverer (v2.2.0.388). Quantities of selected identified peptides were determined in Skyline software (24.1.1.202; University of Washington) based on peak areas in extracted ion chromatograms (EICs), including identification alignment across the raw files based on retention time and *m*/*z*. The mass spectrometry proteomics data have been deposited to the ProteomeXchange Consortium via the PRIDE^20^ partner repository with the data set identifier PXD059514. The quantitative data comparison between preparation approaches was performed using the KNIME Analytics Platform. The percentage representation of a peptide form was derived from the values of EIC precursor peak area as a proportion of the sum of the EIC areas of all forms of the respective peptide sequence (hereafter referred to as “relative abundance”).^21^ The details are provided in the Supporting Information.

## Results and Discussion

### Experimental Rationale and Design

The considerable complexity of histone proteins represents a significant challenge in characterizing them using different methodological approaches.^22,23^ Among the methodologies, MS, with its high throughput, accuracy, and flexibility, is the most suitable strategy for the comprehensive analysis of histone sequence variants and their PTMs.^22,24^ Although protocols for bottom-up histone proteomics are available, several potential pitfalls still need to be addressed at each step of the process.^25^ In our previous work, we established TMA-based derivatization prior to bottom-up LC–MS/MS analysis of histones to facilitate the quantification of histone marks using MS1-level spectral information.^13^ In addition to blocking lysines for trypsin digestion, TMA imparts a higher hydrophobicity to peptides than the commonly used propionic anhydride. Improved chromatographic retention and separation of peptides, including positional isomers, increased the number of identified and quantified forms.^26,27^ Although beneficial, chemical derivatization of histones using TMA at the protein level (i.e., labeling of lysines' amine groups) requires a long incubation time and stepwise addition of TMA to achieve high labeling efficiency. In this regard, we tested whether protein-level derivatization followed by trypsin digestion can be replaced by the specific cleavage of the C-terminal side of arginine residues by the recently introduced Arg-C Ultra proteinase. The performance of Arg-C Ultra for histone digestion was proved by comparison with the Arg-C enzyme and cleavage with trypsin after TMA derivatization (hereafter referred to as “TMA-dT-TMA”). In addition, we evaluated the necessity of derivatization at the peptide level following Arg-C Ultra cleavage (Arg-C Ultra-TMA). For all conditions tested (Figure 1), histones were prepared from the chronic lymphocytic leukemia MEC-1 cell line using extraction into sulfuric acid followed by precipitation with TCA. The number of technical replicates was set to three for all conditions.

**Figure 1:**
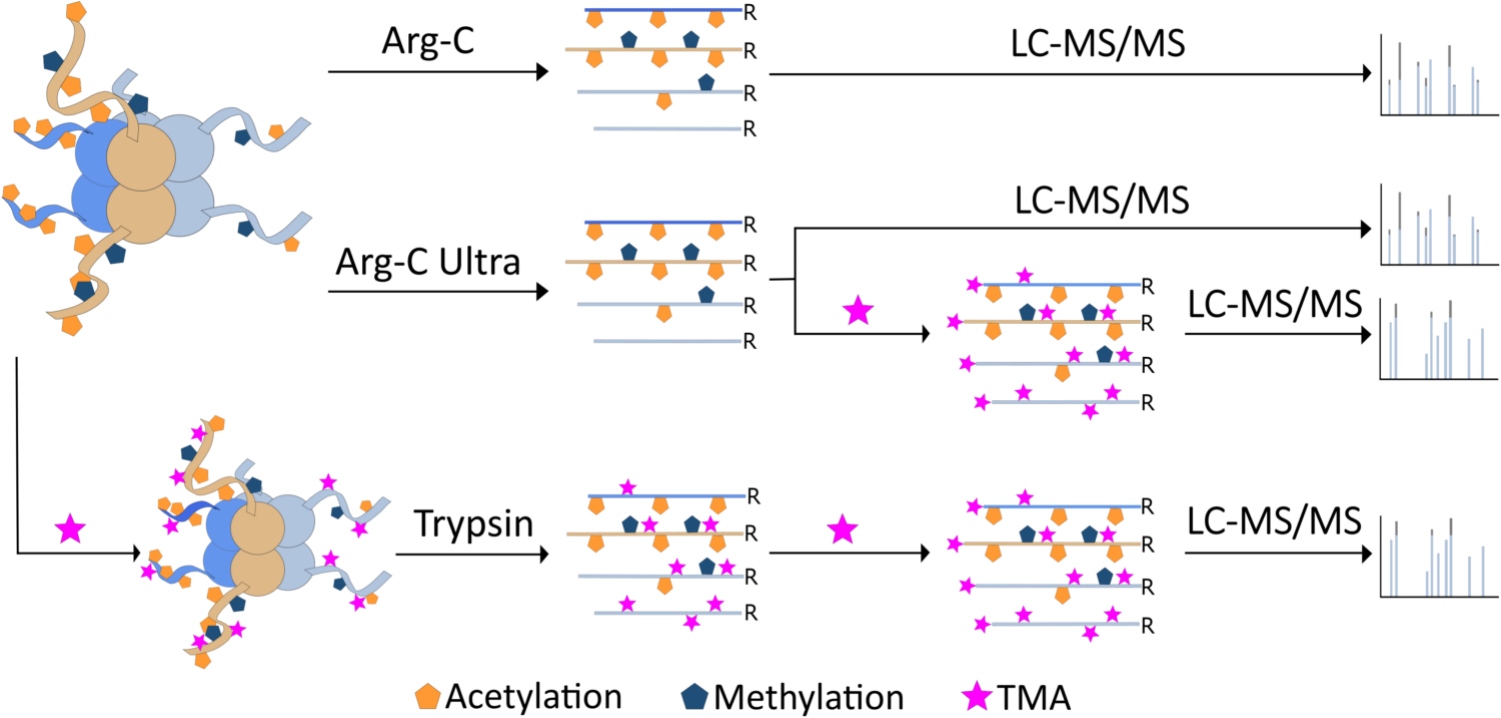
Experimental design. The performance of Arg-C Ultra was compared to Arg-C. Further, the impact of TMA-labeling of peptide forms after Arg-C Ultra cleavage was evaluated and compared with the results of the standard TMA-dT-TMA procedure. The time needed for sample preparation (excluding desalting) is as follows: < 3, < 3, < 4, and ∼72 h for Arg-C, Arg-C Ultra, Arg-C Ultra-TMA, and TMA-dT-TMA, respectively.

### A Comparison of Arg-C Ultra Performance with Other Arginine-Specific Cleavage Procedures

We identified 33 and 36 histone sequence variants in Arg-C and Arg-C Ultra samples, whereas 30 and 29 histone variants were found in Arg-C Ultra-TMA and TMA-dT-TMA samples, respectively (Figure 2A and Supporting Information, Table S1). Compared to Arg-C and Arg-C Ultra, a lower median sequence coverage of histones was achieved in TMA-labeled samples due to the loss of long hydrophobic peptides (Figure 2A). This is demonstrated by the box plots and bar charts depicting different distribution of long peptides with a high number of lysines and shorter peptides with a lower number of lysines in their sequences compared with unlabeled samples. Peptides with a length longer than 14 amino acids (aa) and number of lysines greater than 3 (i.e., values of upper quartiles (*Q*3) in Arg-C Ultra-TMA samples; Figure 2A and detailed description in Table S1) include 316, 170, 109, and 82 modified forms in Arg-C, Arg-C Ultra, Arg-C Ultra-TMA, and TMA-dT-TMA, respectively. As previously reported, no one-fits-all approach is available for the characterization of all histone sequence variants due to their diversity.^13^ Certain histone variants benefit from the chemical derivatization of amine residues, while those in which Arg-C-based cleavage leads to excessively long peptides with a higher number of lysines do not. Due to incomplete fragment ion series, the determination of combinatorial patterns of multiple modified forms of long peptides is problematic in labeled samples. Besides, it is often difficult to achieve 100% conversion of multiple lysines in long peptides, which leads to nondesired peptides after cleavage. In this regard, digestion with Arg-C Ultra without derivatization gave the best results for 29 aa long P1–R29 peptide of H2B type 1-K (UniProt accession number O60814) containing ten lysines, as three correctly digested forms (nonmodified and monoacetylated K12ac and K15ac forms) were identified. On the other hand, short histone forms without labeling have charged lysines and lack sufficient hydrophobicity to be retained or separated at higher resolution by reversed-phase nano-HPLC. For instance, the histone H3 T3KQTAR8 peptide carrying the K4 methylation site, one of the most studied potential epitherapeutic targets for cancer treatment,^28^ was not detected in Arg-C Ultra samples, and only its longer forms with missed cleavages but without modified K4 were identified in Arg-C samples (i.e., ARTKQTARKSTGGKAPR, TKQTARKSTGGKAPR, TKQTARKSTGKAPRKQLATKAAR). The desired T3KQTAR8 peptides containing acetylated, mono-, di-, and trimethylated H3K4 marks were detected only in Arg-C Ultra-TMA and TMA-dT-TMA samples, indicating that TMA labeling, similar to the hybrid chemical derivatization of Prop-PIC,^12^ ensures better retention and detection of hydrophilic peptides during LC-MS/MS. Similarly, TMA labeling enabled quantification of more post-translationally modified forms of G4KQGGKAR11 (G4–R11) peptide of H2A type 3 (UniProt accession number Q7L7L0). Only one nondesired diacetylated form (i.e., SGRGKQGGKAR) of this peptide was identified in Arg-C. In Arg-C Ultra, mono- (K5ac) and diacetylated (K5acK9ac) forms were identified, but the nonmodified form was absent. Increased hydrophobicity by TMA-labeling enabled the identification of nonmodified, mono- (K5ac, K9ac), and diacetylated (K5acK9ac) forms. Benefit of chemical derivatization is also evident from the higher number of identified peptide forms below the median values (*Q*2) of peptide length and number of lysines in Arg-C Ultra-TMA samples (Figure 2A and detailed description in Table S1). Peptides with lengths less than 11 amino acids and number of lysines less than 2 include 101, 120, 177, and 170 modified forms in Arg-C, Arg-C Ultra, Arg-C Ultra-TMA, and TMA-dT-TMA, respectively (Figure 2A and Table S1). Evaluation of the percentage of desired peptide sequences revealed very low cleavage specificity of Arg-C for histones H3.1 and H4 (UniProt accession numbers P68431 and P62805) because they were identified primarily on the basis of nondesired sequences, i.e., nonspecifically cleaved peptides or peptides with missed cleavages (Figures 2B and Supporting Information, S1 and Table S1). Arg-C Ultra, similar to the previously reported arginine-specific proteinase GingisREX (GRX; Genovis, Lund, Sweden),^15^ was shown to cleave specifically at arginine residues, giving more than 97% of desired peptide sequences (Figure 2B). TMA labeling greatly improves the chromatographic separation, including the separation of isobaric peptides (Figure 3), as evidenced by the higher number of identified and quantified post-translationally modified histone peptide forms. Each TMA group increased the difference in retention time on the Aurora Ultimate C18 UHPLC column under the given conditions by between 22 and 24 min compared to the unlabeled samples. Complete trimethylacetylation of naturally diacetylated histone H4 N-terminal peptides (H4 G4–R17) increased retention time by 71 min (Figure 3A). Here, we show the impact of enzymatic digestion and TMA labeling on quantifying histones H3 and H4, for which arginine-specific cleavage is particularly advantageous. Only 44 peptidoforms of H3 and H4 were quantified in Arg-C samples, with 2 forms derived specifically from histone H3.3 (Figure 3B and Supporting Information, Table S2).

**Figure 2:**
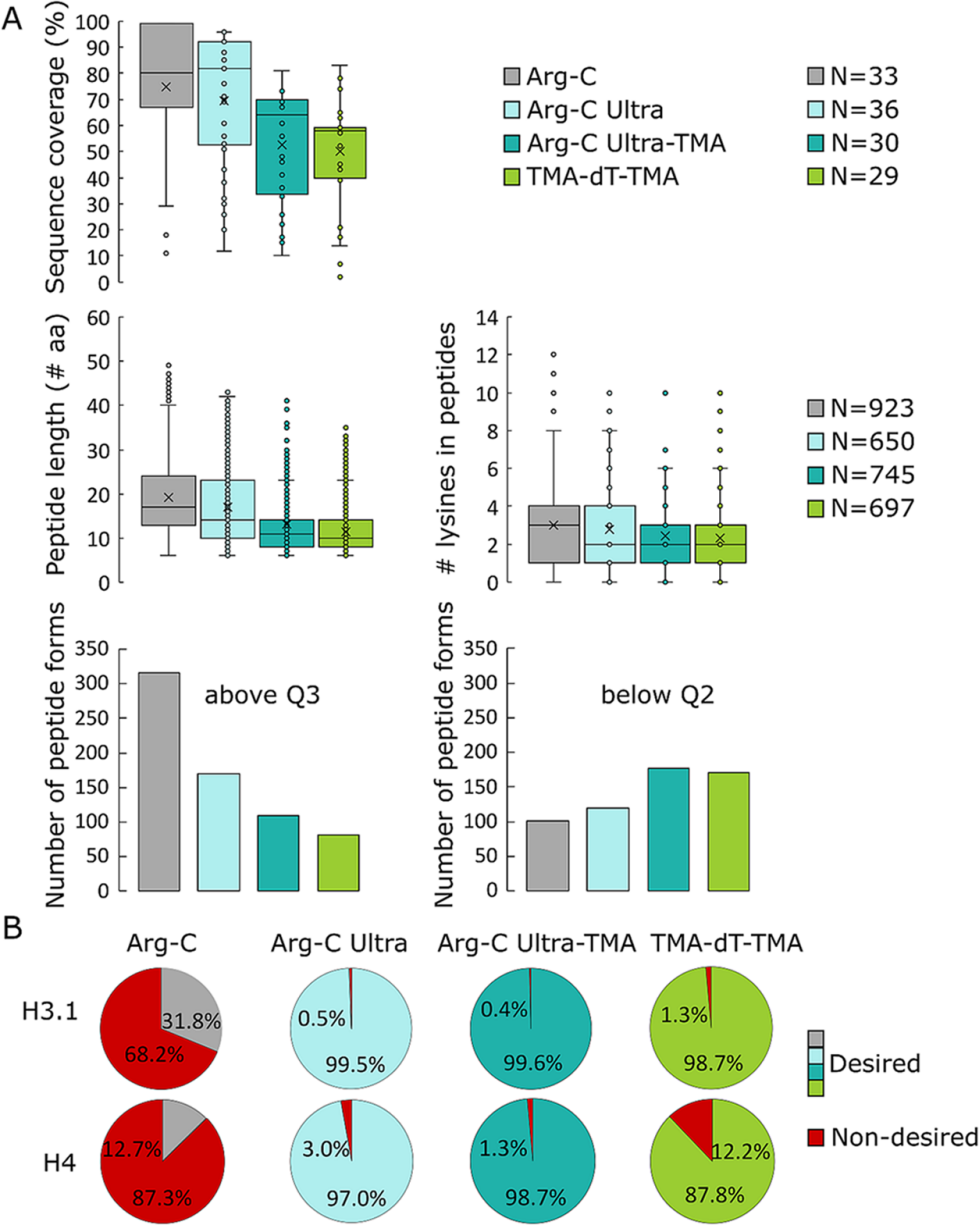
Comparison of histone digestion using Arg-C, Arg-C Ultra, and trypsin. (A) Box-plots of (i) sequence coverage of identified histone variants (*N* – number of histone variants), (ii) peptide length corresponding to number of amino acids (# aa), and number of lysines (#) in histone peptide forms (*N* – total number of peptide groups of all histones). Box plots show extremes, interquartile ranges, and medians. The medians and upper quartiles of peptide length and # lysines in Arg-C Ultra-TMA samples were used as cutoff values for bar graphs showing the benefit of TMA labeling for short hydrophilic peptides but not for long hydrophobic ones (*Q*2—length less than 11 aa and # lysines less than 2, *Q*3—length greater than 14 aa and # lysines greater than 3). (B) Specificity of the digestion, i.e., percentage of desired (peptides cleaved after arginine as expected) and nondesired sequences (nonspecifically cleaved peptides or peptides with missed cleavages—for details, see Supporting data, Figure S1). The data represent median values calculated from three technical replicates for each condition.

**Figure 3:**
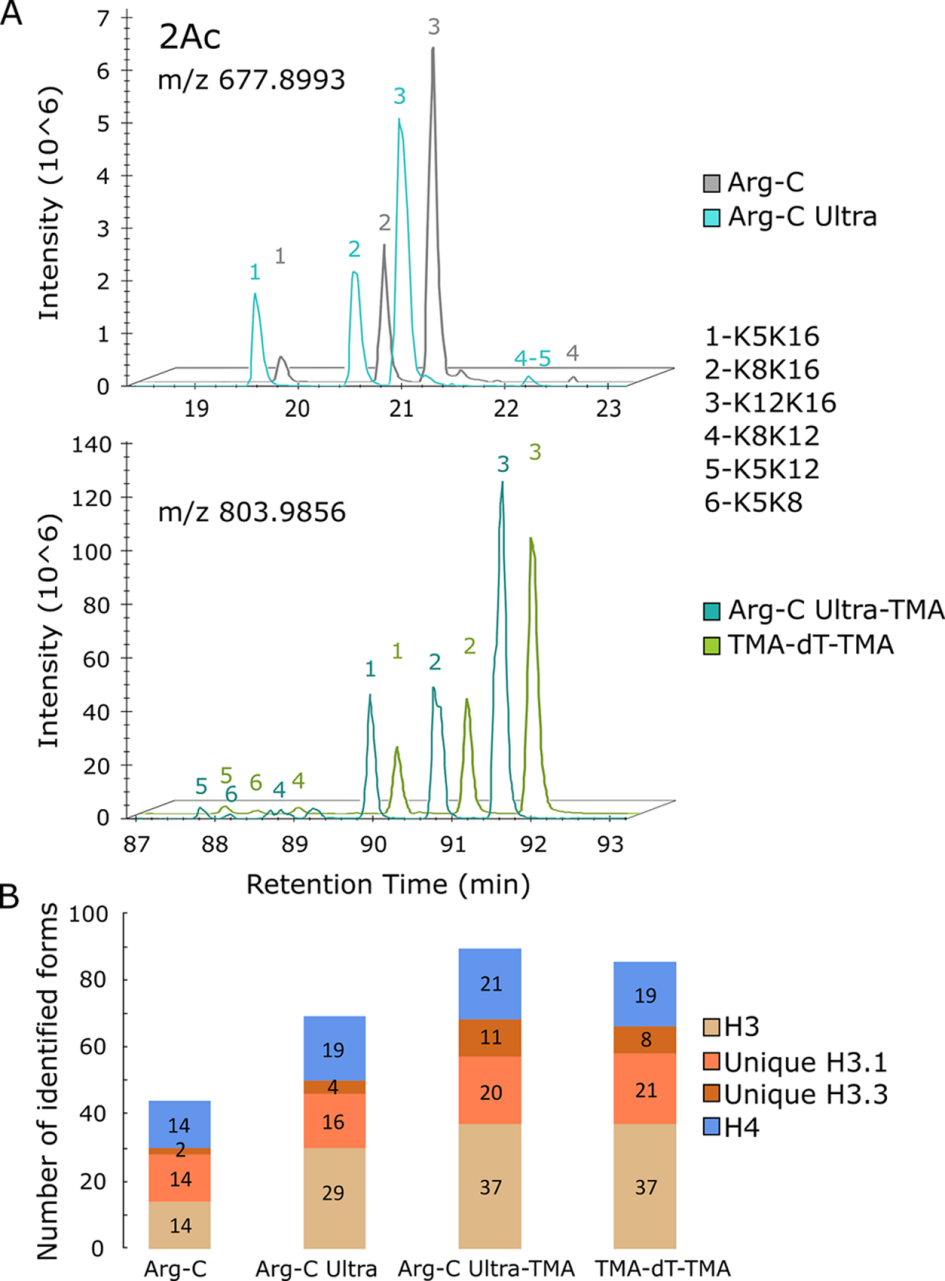
Impact of chromatographic behavior of peptides after Arg-C, Arg-C Ultra, and trypsin digestion with or without chemical derivatization of amine residues on the quantification of post-translationally modified forms. (A) Representative chromatograms of positional isomers of diacetylated N-terminal H4 G4–R17 peptide forms. Lysines carrying acetylation identified in particular peaks of precursor ions in the EIC are indicated. (B) Number of identified and quantified H3.1, H3.3, and H4 histone peptide forms. Peptides identical in H3.1 and H3.3 form a common column, H3.

In Arg-C Ultra, 68 histone forms with 4 sequences specific for histone H3.3 were quantified. In Arg-C Ultra-TMA and TMA-dT-TMA samples, a total of 89 and 85 histone peptide forms, including 11 and 8 peptide forms originating specifically from H3.3, were quantified, respectively. For instance, TMA-enhanced separation of mono-, di-, and triacetylated positional isomers of N-terminal H4 G4–R17 sequences increased the number of quantified forms at the MS1 level from 10 to 15 (Figure 4). Further, TMA-labeling increased the number of identified phosphorylated H3 or acetylated H2A peptidoforms (Figure 4). The benefit of TMA labeling to recover more peptidoforms is reflected in the majority of H3 and H4 peptides carrying PTMs. The neutralization of the positive charge of amine groups, in conjunction with the hydrophobicity increase resulting from the labeling of histone peptides, confers advantages in terms of chromatographic retention of peptides, the separation of isobaric peptides, and the number of identified post-translationally modified forms that can be quantified at MS1 level. By comparing the average log2 transformed areas of the peptide precursors, we show that the sample preparation method significantly affects the quantitation levels (Supporting Information, Figure S2 and Table S2). The lowest precursor areas were found in Arg-C samples; specifically, the median value of precursor areas was almost 2.6 times lower compared to that of Arg-C Ultra. Lower labeling efficiency at the peptide level was detected in Arg-C Ultra-TMA samples, suggesting that an additional derivatization round would be beneficial. Nevertheless, this did not affect the quantitative data as all of the desired Arg-C-like peptides (including those with multiple charge states or incomplete derivatization) were included in the quantitative evaluation by summing the areas of the relevant precursor peaks in the KNIME platform. The median precursor area in Arg-C Ultra-TMA samples was three times higher compared to TMA-dT-TMA. This may reflect the high specificity of the Arg-C Ultra cleavage at arginine residues as well as certain losses due to insufficient labeling at the protein level in TMA-dT-TMA samples (as shown in Figure 2B). However, the correlation between relative values of peak areas of precursor ions was high between conditions (Figure S3). The very high correlation between Arg-C Ultra-TMA and TMA-dT-TMA (SCC = 0.92) confirms that both approaches are comparable and represent alternatives for quantifying post-translationally modified histone peptide forms. Both approaches also showed comparable standard deviations of precursor areas (Supporting Information, Figure S3 and Table S2).

**Figure 4:**
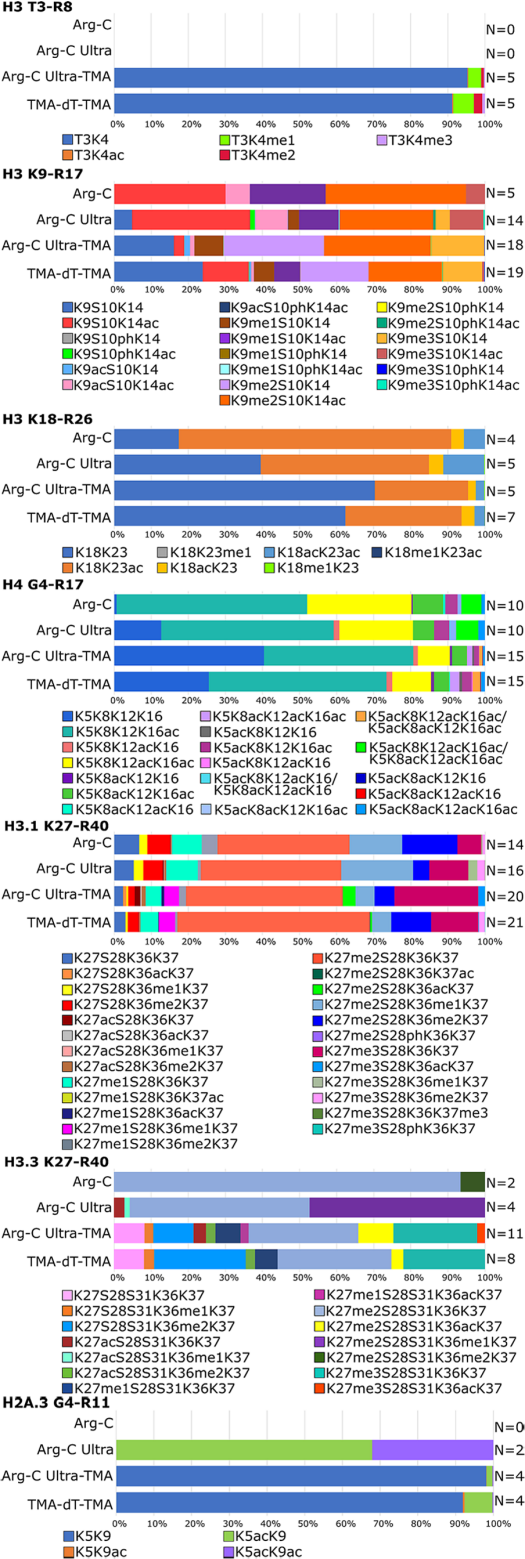
Relative abundance of the identified post-translationally modified N-terminal forms of histone H3, H4, and H2A.3 after Arg-C, Arg-C Ultra, and trypsin digestion with or without chemical derivatization of amine residues. Relative abundance corresponds to the percentage of individual peptide form peak area of EIC from the sum of the EIC areas of all forms of the respective peptide sequence. The number (*N*) of quantified forms observed under each condition is indicated on the right. The legend columns follow the order of the peptide forms in the bar chart from left to right.

## Conclusions

In summary, we show that the novel Arg-C Ultra proteinase with increased arginine cleavage specificity facilitates histone preparation for LC-MS/MS. The elimination of protein-level derivatization substantially simplifies the protocol, reduces the time for sample preparation, and prevents the formation of nonspecific peptides due to insufficient labeling. It allows either direct quantification of long peptides with multiple lysines or, increasingly advantageous for most histone peptides, the use of a wide range of compounds for chemical derivatization of amine groups, which was previously not possible due to their low labeling efficiency at the protein level. Thus, Arg-C Ultra cleavage followed by labeling exclusively at the peptide level opens up the possibility of further improving the chromatographic separation and subsequent identification and quantification of histone peptidoforms.

## Supplementary Material







## Notes

Detailed experimental section, including materials and reagents; Specificity of the digestion using Arg-C, Arg-C Ultra, and trypsin (Figure S1); Overview of quantitative results obtained using different conditions for preparing histone peptides (Figure S2); Scatter plots – means relative abundance values (%) (Figure S3) (PDF)
Identified histone sequence variants; Specificity of the digestion using Arg-C, Arg-C Ultra, and trypsin; Peptide length and number of lysines in the sequences (XLSX)
Relative and absolute abundances of identified peptide forms (XLSX)
