# Supplementary material for: Arg‑C Ultra Simplifies Histone Preparation for LC-MS/MS
Source: Anal Chem. 2025 Jun 12;97(24):12486–92. doi: 10.1021/acs.analchem.5c02238 (PMC12199229; doi:10.1021/acs.analchem.5c02238)
Supplement: Supplementary file 1 [file ac5c02238_si_001.pdf]

Supporting Information

Arg-C Ultra simplifies histone preparation for LC-MS/MS

Palina Ryzhaya[1,2], Pavlína Pírek[1], Zbyněk Zdráhal[1,2], Gabriela Lochmanová[1,2*]

1. Central European Institute of Technology, Masaryk University, 625 00 Brno, Czech Republic
2. National Centre for Biomolecular Research, Faculty of Science, Masaryk University, 625 00 Brno, Czech Republic

ABSTRACT: Arginine-specific cleavage is the primary method used to prepare lysine-rich histone proteins in bottom-up proteomics. As the Arg-C enzyme has demonstrated suboptimal specificity, cleavage at the carboxyl side of arginine residues is typically achieved through chemical derivatization of lysines followed by trypsin digestion. Recent improvements in proteolytic enzymes are reflected in the introduction of Arg-C Ultra, a recombinant proteinase with substantially improved digestion specificity. Here, using mammalian histone extract, we demonstrate that Arg-C Ultra facilitates histone preparation for LC-MS/MS. We show the performance of Arg-C Ultra in terms of digestion specificity, number of modified forms identified, and yield of quantitative information, compared with Arg-C and trypsin digestion combined with chemical derivatization with trimethylacetic anhydride. Importantly, we show that chemical derivatization at the peptide level, *i.e.*, after Arg-C Ultra digestion, is still necessary to improve the quantification of short histone peptidoforms as well as positional isomers.

***Corresponding Author**

gabriela.lochmanova@ceitec.muni.cz
orcid: 0000-0002-0490-9380

Table of Contents

Experimental section 2

1. Materials and Reagents 2
2. Preparation of histone extracts from human cell culture 2
3. Sample desalting 2
4. LC-MS/MS analysis 2
5. Database search, data evaluation, and statistical analysis 2

Supporting results 3

1. A comparison of Arg-C Ultra performance with other arginine-specific cleavage procedures 3

Supporting References 4

Experimental section

1. Materials and Reagents

Iscove's modified Dulbecco's medium, phenol red, fetal bovine serum, L-glutamine, and phenylmethylsulfonyl fluoride (PMSF) were purchased from Thermo Fisher Scientific (MA, USA). Ethylenediaminetetraacetic acid (EDTA) and Bradford assay were purchased from Bio-Rad (CA, USA). Triton X-100 was purchased from Carl Roth (Germany). Sulfuric and hydrochloric acids were purchased from Penta (Czech Republic). Acetonitrile (ACN) and formic acid (FA) were purchased from Honeywell (NC, USA). Trimethylacetic anhydride (TMA), trichloroacetic acid (TCA), ammonium bicarbonate (AB), and dithiothreitol (DTT) were purchased from Merck (Germany). AttractSPE® Tips C18 were purchased from Affinisep (France).

2. Preparation of histone extracts from human cell culture

MEC-1 chronic lymphocytic leukemia cell line (DSMZ no.: ACC 497; German Collection of Microorganisms and Cell Cultures GmbH) was used for histone preparation. The cell line was cultured in Iscove's modified Dulbecco's medium, consisting of phenol red, 10% (v/v) fetal bovine serum, 2mM L-glutamine, and 100 IU penicillin/streptomycin, at 37 °C with 5% CO_2. Once the cells had reached confluence, they were harvested, and histones were extracted following the established protocol[1]. In summary, after two washes with ice-cold PBS, the cells were incubated in a lysis buffer (80mM NaCl, 20mM EDTA, 1% Triton X-100, 45mM sodium butyrate, and 0.1mM PMSF) for 20 min on ice. The cell lysate was centrifuged at 2,000 *g* for 8 min. Histones were extracted from the chromatin pellet into 250 μL of ice-cold 0.2M H_2SO_4 for 2 h at 4 °C. The supernatant was cleared by centrifugation at 16,000 *g* and 4 °C for 8 min, then mixed with 250 μL of 50% ice-cold trichloroacetic acid and incubated with shaking at 0 °C for 30 min. The precipitate was collected by centrifugation at 5,000 *g* and 4 °C for 30 min, washed with 50mM HCl in acetone, twice with acetone, and dried at room temperature. Histone extract was dissolved in water, and protein concentration was determined using the Bradford assay.

3. Sample desalting

The dried samples were reconstituted in 50 μL of 0.1% trifluoroacetic acid (TFA). Desalting was performed using AttractSPE® Tips C18. Peptides were sequentially eluted with 2×10 μL of 0.1% TFA in 50% ACN and 2×20 μL of 0.1% TFA in 75% ACN. The pooled eluates were transferred to an LC vial, concentrated to 10 μL, and acidified with FA to a final concentration of 1 %.

4. LC-MS/MS analysis

The samples were analyzed in a random order using an Ultimate 3000 RSLCnano liquid chromatograph coupled to an Orbitrap Fusion Lumos Tribrid mass spectrometer (Thermo Fisher Scientific). The 1.5 μL of each peptide mixture was injected, concentrated on an online trap column (cartridge type μPrecolumn, 300 μm ID, 5 mm long, Thermo Fisher Scientific) packed with C18 PepMap100 (5μm particles, 100 A), and separated on an Aurora C18 analytical column (25 cm long, 75 μm inner diameter, 1.7 μm particles; Ion Opticks, Australia). Trap and analytical columns were tempered at 25 °C and at 50 °C, respectively. The mobile phases used for the gradient elution consisted of a binary mixture of 0.1% FA in water (A) and 0.1% FA in 80% ACN (B). Peptides were eluted with a 120min gradient at a 300 $nL.min^{-1}$ flow rate and the content of B rising from 3 % (0–4 min), 3 to 42 % (4–107 min), 42 to 80 % (107–115 min), and followed by an isocratic wash of 80 % B (115–120 min). Before sample injection into the loop, the trapping and analytical columns were equilibrated with 99:1 (mobile phase A:B) at a 400 nL/min flow rate. The analytical column outlet was directly connected to the Digital PicoView 550 ion source equipped with Active Background Ion Reduction Device (ESI Source Solutions, Woburn, MA, USA).

Mass spectra were acquired in data-dependent acquisition (DDA) mode using 350 to 2000 m/z survey scans at a resolution of 120,000 (at m/z 200) with an automatic gain control target setting of 4×10^5 and maximum injection time of 500 ms. Precursors with charge states from 2+ to 7+ and intensity above 1×10^4 were subjected to higher energy collisional dissociation fragmentation with normalized collision energy of 30 %. Once fragmented, precursors were excluded for 30 s before the next fragmentation. Precursors were isolated by quadrupole with a 1.2 m/z isolation window. Tandem mass spectra were obtained using 30,000 resolution (at m/z 200). Ions were accumulated for a target value of 5×10^4 or 500 ms injection time. The cycle time between master scans was 2.5 s.

5. Database search, data evaluation, and statistical analysis

Raw data acquired in DDA mode were analyzed against several databases, including the modified cRAP contamination database (based on http://www.thegpm.org/crap/; 112 sequences), an in-house histone human database (v191011; 53 protein sequences generated from UniProt), and the UniProt KB Human database (v240724; taxon ID: 9606; 20,654 sequences). The searches were conducted using the in-house Mascot search engine (v2.6.2; Matrix Science, MA, USA) via Proteome Discoverer software (v2.2.0.388). The mass error tolerance for precursor ions was set at 10 ppm for all database searches. For MS/MS fragment ions, the tolerances were 0.5 Da for the cRAP and UniProt KB Human databases and 0.03 Da for the histone human database. Enzyme specificity was configured to semi-Arg-C, allowing for two missed cleavages across all databases. Variable modifications for each database were as follows: cRAP − acetylation (protein N-terminal region), deamidation (N and Q), oxidation (M), and trimethylacetylation (peptide N-terminal region, K); Histone Human − acetylation (K), phosphorylation (S, T), methylation (K, R), dimethylation (K), trimethylation (K), and trimethylacetylation (peptide N-terminal region, K, S, T, and Y); UniProt KB Human database − trimethylacetylation (peptide N-terminal region, K). For underivatized sample analyses, the same modifications were used except for trimethylacetylation. Peptides identified using the in-house human histone database were

refined by fixed-value peptide-spectrum match validator (delta Cn < 0.05), followed by filtering highly confident peptides using Mascot parameters set to Rank 1, expectation value <0.01, and ion score ≥ 30. Peptides identified using the UniProt KB Human database were refined by Percolator (Cn < 0.05, false discovery rate < 0.01). Selected identified peptides were manually inspected, and their quantities were determined and manually validated using Skyline-daily software (24.1.1.202; University of Washington) based on peak areas in extracted ion chromatograms (EICs), including identification alignment across the raw files based on retention time and m/z.

The quantitative data comparison between sample preparation approaches (Arg-C, Arg-C Ultra, Arg-C Ultra-TMA, and TMA-dT-TMA) was performed using the KNIME Analytics Platform with R scripts. The precursor peak areas in EIC of histone peptide forms were log2 transformed. The areas of peptide forms present in multiple charge states or incompletely derivatized forms in the EIC were summed to obtain overall peptide form abundance. The means and standard deviations of peptide abundances for each digestion method were calculated and compared using Student's t-tests. The relative abundances of histone modified peptide forms were calculated as the ratio of each individual EIC precursor peak area to the sum of the EIC areas of all forms of respective peptide sequence. Peak areas for all forms were treated as compositions and combined using geometric means.[2] To compare mean relative abundance of each peptide between sample preparation approaches, Spearman's correlation analysis was performed.

Supporting results

1. A comparison of Arg-C Ultra performance with other arginine-specific cleavage procedures

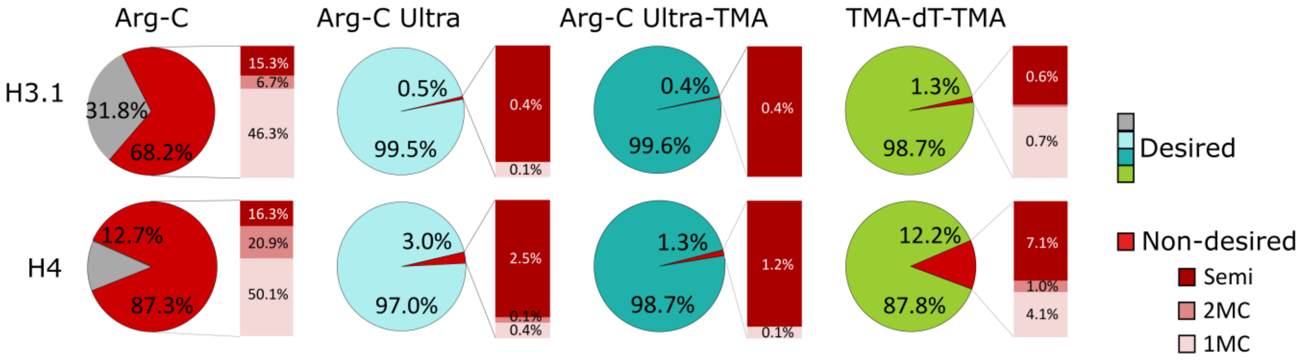

Figure S1. Specificity of the digestion using Arg-C, Arg-C Ultra, and trypsin. Percentage of desired (peptides cleaved after arginine as expected) and non-desired sequences comprising of non-specifically cleaved peptides (Semi) or peptides with one (1MC) or two (2MC) missed cleavages are shown. The data represent median values calculated from three technical replicates for each condition.

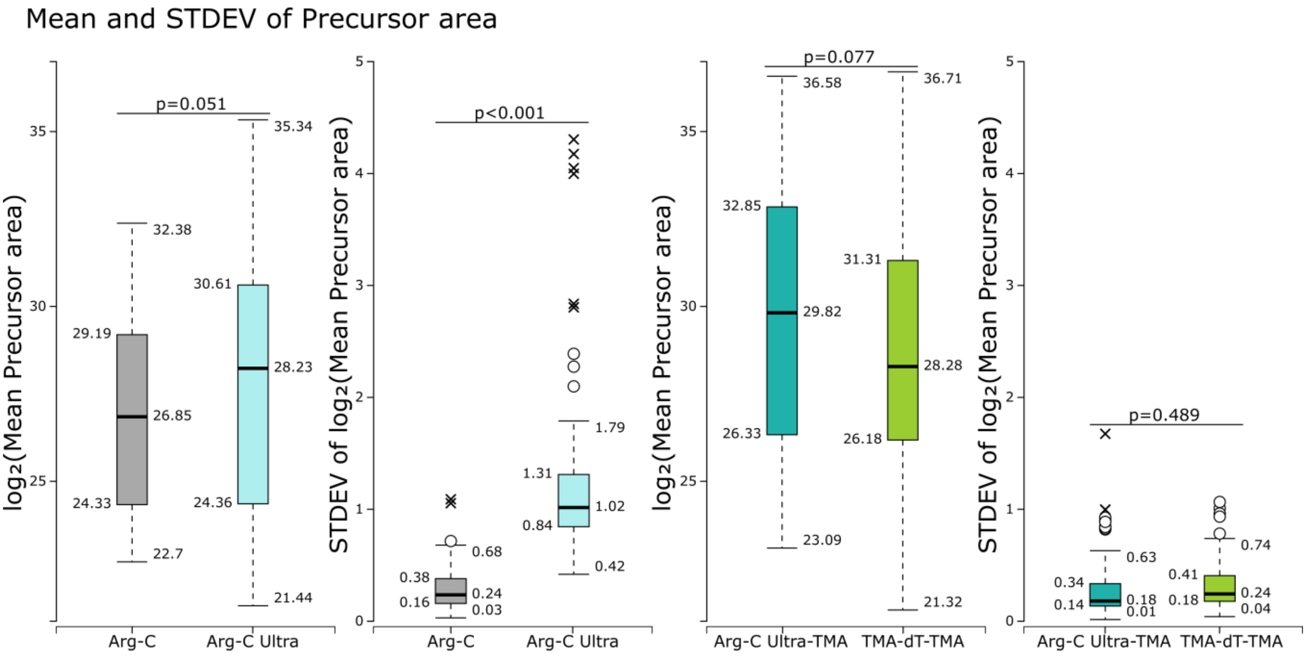

Figure S2. Overview of quantitative results obtained using different conditions for preparing histone peptides. Comparison of means and standard deviations (STDEV) of histone H3 and H4 precursor areas. The box plots show extremes, interquartile ranges, and medians (N = 44, 68, 89, and 85 for Arg-C, Arg-C Ultra, Arg-C Ultra-TMA, and TMA-dT-TMA, respectively). Means and standard deviations were compared by Student’s t-tests (p-values).

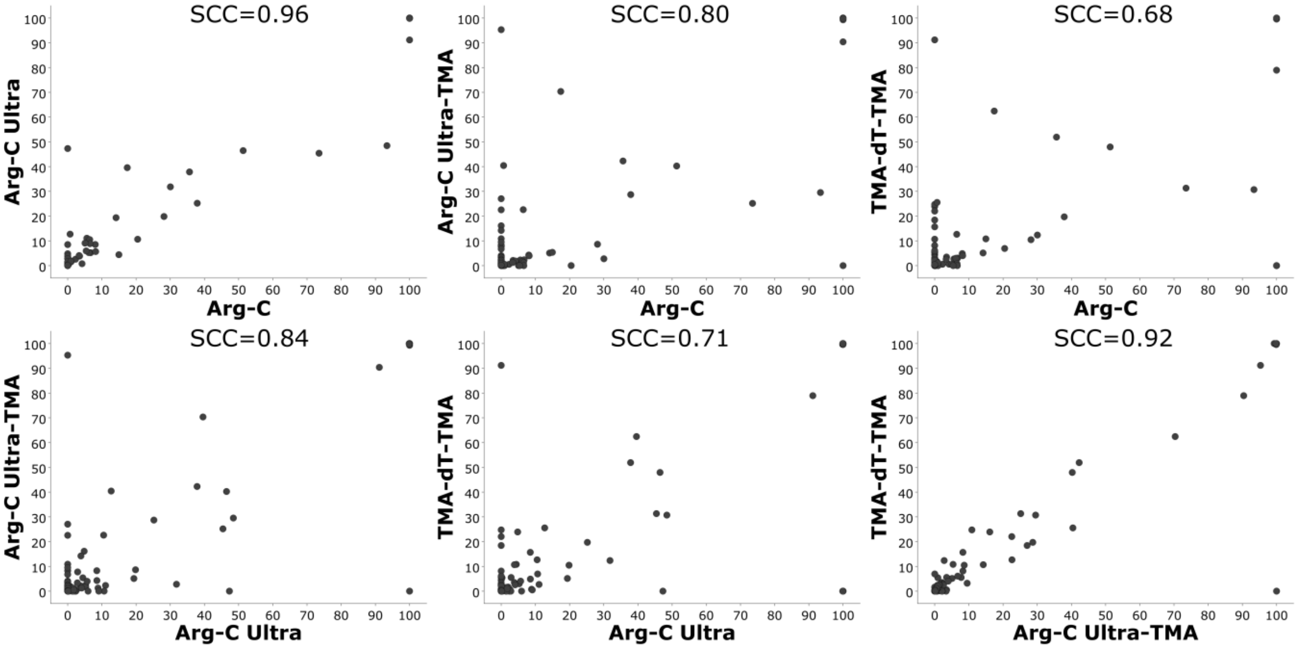

Figure S3. Scatter plots – means relative abundance values (%). Means were compared by Spearman's correlation coefficients (SCC values). The data represent median values calculated from three replicates for each condition.

Supporting References

(1) Činčárová, L.; Lochmanová, G.; Nováková, K.; Šultesová, P.; Konečná, H.; Fajkusová, L.; Fajkus, J.; Zdráhal, Z. A Combined Approach for the Study of Histone Deacetylase Inhibitors. *Mol Biosyst* **2012**, *8* (11), 2937–2945. https://doi.org/10.1039/c2mb25136a.

(2) Lochmanová, G.; Ihnatová, I.; Kuchaříková, H.; Brabencová, S.; Zachová, D.; Fajkus, J.; Zdráhal, Z.; Fojtová, M. Different Modes of Action of Genetic and Chemical Downregulation of Histone Deacetylases with Respect to Plant Development and Histone Modifications. *Int J Mol Sci* **2019**, *20* (20), 5093. https://doi.org/10.3390/ijms20205093.
